# The increase of vasomotor tone avoids the ability of the dynamic preload indicators to estimate fluid responsiveness

**DOI:** 10.1186/1471-2253-13-41

**Published:** 2013-11-11

**Authors:** Juan P Bouchacourt, Juan A Riva, Juan C Grignola

**Affiliations:** 1 Department of Anesthesia, Hospital de Clínicas, Facultad de Medicina, Universidad de la República, Avda Italia 2870, PC 11600, Montevideo, Uruguay; 2 Department of Pathophysiology, Hospital de Clínicas, Facultad de Medicina, Universidad de la República, Avda Italia 2870, PC 11600, Montevideo, Uruguay

**Keywords:** Polar plot, Preload dynamic indices, Pulse pressure variation, Stroke volume variation, Vasomotor tone, Phenylephrine

## Abstract

**Background:**

The use of vasoconstrictor can affect the dynamic indices to predict fluid responsiveness. We investigate the effects of an increase of vascular tone on dynamic variables of fluid responsiveness in a rabbit model of hemorrhage, and to examine the ability of the arterial pressure surrogates dynamic indices to track systolic volume variation (SVV) during hypovolemia under increased vasomotor tone.

**Methods:**

Eighteen anesthetized and mechanically ventilated rabbits were studied during normovolemia (BL) and after blood progressive removal (15 mL/kg, BW). Other two sets of data were obtained during PHE infusion with normovolemia (BL + PHE) and during hypovolemia (BW + PHE). We measured central venous and left ventricular (LV) pressures and infra diaphragmatic aortic blood flow (AoF) and pressure. Pulse pressure variation (PPV), systolic pressure variation (SPV) and SVV were estimated manually by the variation of beat-to-beat PP, SP and SV, respectively. We also calculated PPV_apnea_ as 100 × (PP_max_-PP_min_)/PP during apnea. The vasomotor tone was estimated by total peripheral resistance (TPR = mean aortic pressure/mean AoF), dynamic arterial elastance (Ea_dyn_ = PPV/SVV) and arterial compliance (C = SV/PP). We assessed LV preload by LV end-diastolic pressure (LVEDP). We compared the trending abilities between SVV and pressure surrogate indices using four-quadrant plots and polar plots.

**Results:**

Baseline PPV, SPV, PPV_apnea_, and SVV increased significantly during hemorrhage, with a decrease of AoF (*P* < 0.05). PHE induced significant TPR and Ea_dyn_ increase and C decrease in bled animals, and a further decrease in AoF with a significant decrease of all dynamic indices. There was a significant correlation between SVV and PPV, PPV_apnea_ and SPV in normal vasomotor tone (r^2^ ≥ 0.5). The concordance rate was 91%, 95% and 76% between SVV and PPV, PPV_apnea_ and SPV, respectively, in accordance with the polar plot analysis. During PHE infusion, there was no correlation between SVV and its surrogates, and both four-quadrant plot and polar plot showed poor trending.

**Conclusion:**

In this animal model of hemorrhage and increased vasomotor tone induced by phenylephrine the ability of dynamic indices to predict fluid responsiveness seems to be impaired, masking the true fluid loss. Moreover, the arterial pressure surrogates have not the reliable trending ability against SVV.

## Background

Fluid resuscitation protocols to optimize preload and cardiac output (CO) in patients undergoing high-risk surgery decreased hospital length of stay and postoperative complications [1,2]. While hypovolemia (under-resuscitation) can result in organ hypoperfusion and ischemia, over-resuscitation (inadequate fluid administration) can result in pulmonary and interstitial edema and increase the morbidity and mortality of critically ill patients [3,4].

Based on the heart-lung interplay during mechanical ventilation, fluid optimization has changed from CO optimization (maximization) to monitor dynamic parameters of volume responsiveness [5,6]. These indices monitor the respiratory change in stroke volume (SV) or its surrogates, such as pulse pressure or systolic pressure induced by positive pressure ventilation (functional hemodynamic monitoring) [6]. Cyclic change in intrathoracic blood volume, due to positive-pressure ventilation is the main determinant of the observed SV variation (SVV) and pulse pressure variation (PPV) during mechanical ventilation [7]. The magnitude of the respiratory changes in the left ventricular (LV) SV (SVV) or its arterial pressure surrogates (PPV, the PPV during apnea, -PPV_apnea_-, and the systolic pressure variation, -SPV-) is an indicator of preload reserve [5,6,8-11]. The higher the pre-infusion value of the dynamic indices, the more pronounced the increase in SV in response to fluid administration will be [5]. The threshold values of dynamic variables of fluid responsiveness range from 10 to 15% and a change greater than 12 to 13% (cut-off values) is highly predictive of fluid responsiveness [6].

Several studies have shown excellent accuracy for these dynamic indices under strict conditions [4,11]. However, there are several confounders in routine clinical practice that can significantly reduce the predictive value of SVV and its surrogates of fluid responsiveness, including low tidal volume, cardiac arrhythmias, automatic calculation method, intra-abdominal hypertension, elevated positive end-expiratory pressure and use of vasopressor drugs [5,12]. Since vasoactive drugs alter arterial tone as well as venous capacitance, their use in clinical practice may affect the accuracy of functional preload indicators to assess fluid responsiveness, especially those arterial pressure surrogates. To date, available data are conflicting (either in a controlled experimental study or a clinical study), depending on the vasopressor drug (norepinephrine versus phenylephrine), the device to estimate and track the SV (pulse pressure contour versus doppler flowprobe) and the study conditions (normovolemia versus hypovolemia, hypertension versus hypotension) [13-18].

We hypothesized that the use of a vasoconstrictor drug may attenuate the increase of dynamic indices (SVV, PPV, SPV, PPV_apnea_) during hypovolemia. The first aim of this study was to investigate the effects of an increase of vascular tone on preload dynamic variables of fluid responsiveness in a rabbit model of hemorrhage. The second aim was to examine the ability of the arterial pressure surrogates dynamic indexes to track SVV changes during hypovolemia under increased vasomotor tone.

## Methods

This study was approved by the Institutional Animal Care and Use Committee (CHEA, Facultad de Medicina, Universidad de la República -http://www.chea.udelar.edu.uy/). We rigidly performed all institutional protocols to handle animals under experimentation according to Guide for the Care and Use of Laboratory Animals (NIH Publication N° 85–23, revised 1996), prepared by the National Academy of Sciences’ Institute for Laboratory Animal Research.

### Animal instrumentation

Eighteen female New Zealand rabbits (body weight 2.56 ± 0.56 kg) were premedicated with acepromazine (0.3 mg/kg i.m.) and meperidine (10 mg/kg i.m.). Anesthesia was induced with a bolus dose of midazolam (0.5 mg/kg i.v.) given via an ear vein. We tracheotomized and mechanically ventilated (Amadeus Hamilton Medical AG, Switzerland) the animals via an endotracheal tube (ID 2.5 mm). The ventilator was set in the volume controlled ventilation mode with a tidal volume of 9 mL/kg, end-expiratory pressure of 5 cmH_2_O, a respiratory rate 40 ± 8 breaths/min and a FiO_2_ of 60%. Normocapnia, guided by capnography (Datex Inst Corp CD-200-43-00, Helsinki, Finland) and monitored by serial blood gas evaluation (ABL520, Radiometer, Denmark), was achieved by adjusting the respiratory rate to maintain an end-tidal CO_2_ tension between 30–38 mm Hg. Heart rate/respiratory rate ratio was > 3.6. We maintained the anesthesia with a continuous infusion of midazolam (0.5-1 mg/kg/h) to achieve the least and constant effect on peripheral resistance and rocuronium bromide (0.6 mg/kg/h) to avoid spontaneous breathing effort. Intravenous saline solution (NaCl 0.9%) was administered at a rate of 7 mL/kg/h as maintenance requirements [19]. Normothermia was kept using a heating pad.

We placed a 4.5 F triple-lumen central venous catheter (Paediatric Multicath 3-Vygon) in the left jugular vein for measuring central venous pressure, blood withdrawal and drug infusion. A 20-gauge catheter was placed into the LV through the right common carotid to monitor LV pressure. A non constricting ultrasonic perivascular flow probe (2.5PSB-Series Flow probe, Transonic Systems Inc., Ithaca, NY, USA) was positioned around the infra-diaphragmatic aorta by a right lumbar incision by an extra-peritoneum approach and connected to a Doppler flowmeter (model T101, Transonic Systems Inc., Ithaca, NY, USA) to measure instantaneous aortic flow. A fluid-filled catheter (20-gauge) was advanced through the right femoral artery up to the infra-diaphragmatic aorta, just distal to the flow probe to monitor systemic arterial blood pressure. All pressure transducers (P23Db Gould Statham) were zeroed to atmospheric pressure and kept at the atrial level.

### Experimental protocol

Once we completed the instrumentation, the animals were allowed to stabilize for 30 min. Baseline measurements including all the variables described previously were obtained during normovolemia (BL). After BL measurement, the vasomotor tone was increased by phenylephrine infusion (Sigma, St. Louis, MO) during 30 min and a second set of hemodynamic data were obtained (BL + PHE). Taking into account the half-life of PHE, we allowed 20 minutes period after stopping phenylephrine infusion for the rabbits to return to baseline, then, blood was progressively withdrawn by stepwise cumulative volume of 5 mL/kg with a total of 15 mL/kg of body weight (20% of volemia) and a third set of hemodynamic measurements were obtained (BW). Finally, 30 min after BW, a fourth set of data were obtained during PHE administration (BW + PHE). We adjust the PHE infusion rate in order to obtain an increase of the vasomotor tone, maintaining mean arterial pressure around the baseline value (isobaric analysis). This allowed us to avoid an indirect effect of intravascular arterial pressure on the viscoelastic properties of the arterial wall. Once the experimental protocol was completed, the animals were killed with intravenous potassium chloride under deep anesthesia.

### Data collection and analysis

All signals were monitored in real time and stored digitally with a hardware and software specially designed in our laboratory (SAMAY M16) at a sampling frequency of 200 Hz. Hemodynamic data were taken at the end of expiration with the ventilator turned off.

**Figure 1:**
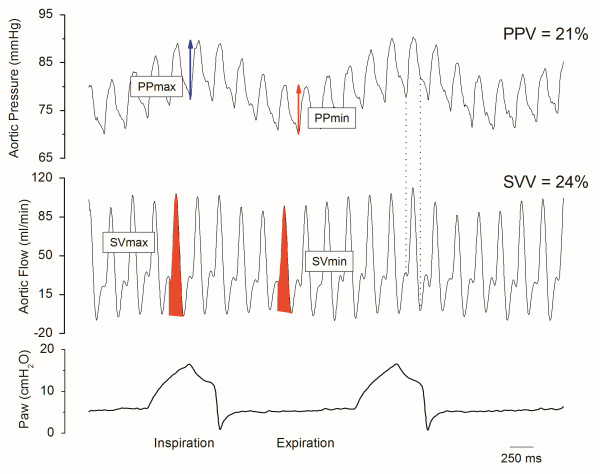
**Raw data showing aortic pressure and flow in one rabbit after blood withdrawal.** PP: pulse pressure, SV: stroke volume, Paw: airway pressure, SVV: stroke volume variation, PPV: pulse pressure variation. Shaded areas represent the SV during expiration (SVmin) and inspiration (SVmax), respectively. Dotted lines state the systolic portion of the aortic flow curve.

SV was derived offline from the integral of the systolic portion of the aortic flow curve for each cardiac cycle (Figure 1). The vasomotor tone was assessed by total arterial peripheral resistance (TPR = mean aortic pressure/mean aortic flow), arterial capacitance (C = SV/arterial pulse pressure) [20], and dynamic arterial elastance (Ea_dyn_ = PPV/SVV) [21]. LV preload was assessed by end-diastolic pressure. The first derivative of LVP (dP/dt) was digitally obtained in order to estimate LV contractility (maximum dP/dt, dP/dtmax) and LV active diastolic function (minimum dP/dt, dP/dtmin). All continuous values were averaged over a 5-sec period. PPV, SVV and SPV were calculated offline. The calculus was performed over three consecutive respiratory cycles including five heartbeats each. Dynamic indices are defined by the relative difference in maximum and minimum values for pulse pressure (PP_max_/PP_min_), systolic pressure (SP_max_/SP_min_), and SV (SV_max_/SV_min_) for PPV, SPV, and SVV, respectively according to:

$$100\;\times \;\left(Q_{\text{max}}-Q_{\text{min}}\right)/\left[\left(Q_{\text{max}}+Q_{\text{min}}\right)/2\right]$$

with Q = PP, SP, SV for PPV, SPV, and SVV, respectively (Figure 1).

Finally, we measured the PP during apnea (PP_apnea_) and used this value to calculate the PPV_apnea_ as: 100 × (PP_max_ - PP_min_)/ PP_apnea_[9].

### Statistical analysis

Statistical comparisons were performed using statistics software (SPSS for Windows Version 18.0; SPSS Inc., Chicago, IL). Normal distribution was tested with the Kolmogorov–Smirnov test. All data are expressed as mean ± SD. Before the start of the study, the number of animals required was determined with a power analysis according to previous data and the target difference. In accordance with a standardized difference (target difference/standard deviation, 11/8) of 1.37, a sample size of 18 animals was calculated for a P value of 0.05 with a power of 80% [22]. PPV, SVV, SPV and PPV_apnea_ were analyzed at the different experimental conditions using one-way analysis of variance for repeated measurements (ANOVA). Post-hoc testing was performed using the Bonferroni test. The relationship between SVV and its surrogates (PPV, PPV_apnea_, SPV) were examined using Pearson correlations. A *P* value < 0.05 was considered statistically significant.

We used four-quadrant scatter plots to compare the concordance rate of SVV and the arterial pressure surrogates indexes (PPV, PPV_apnea_, SPV) during BL and BW (without PHE, PHE-) and during PHE infusion (PHE+). The concordance rate was defined as the percentage of the number of data points that are in two of the four quadrants of agreement (upper right and lower left). We also performed a polar plot analysis to compare the trending abilities between SVV and its pressure surrogates during PHE - and PHE+. Polar plots present the data from a 4-quadrant plot in a similar format to a Bland-Altman plot but with a radial distribution of data points about a polar origin. It allows a narrower and more selective band of agreement to be applied to the data [23]. We calculated the mean angular bias which is the average angle between all polar axes and polar data points, and the radial limits of agreement which is the radial sector containing 95% of the data points. The acceptance limits in the polar plot analysis were an angular bias of less than ± 5° and radial limits of agreement of less than ± 30°. Although there is no guidance on suitable exclusion zones, we applied an exclusion zone when the percentage of change of SVV data was below 15% (0.05) [24].

## Results

Table 1 shows the changes in baseline hemodynamic data during hemorrhage and PHE infusion. Mean doses of PHE infusion during BL + PHE and BW + PHE was 15 ± 2 mcg/kg/min. Hemorrhage (median blood volume loss, 33 ± 7 mL, ≈15 mL/kg) induced a significant decrease of AoF (*P* < 0.05), meanwhile systemic arterial pressure (mean and pulse pressure), C, TPR and Ea_dyn_ did not show a significant change.

**Table 1:** Hemodynamic data during normovolemia (BL), normovolemia with phenylephrine infusion (BL + PHE), hypovolemia (BW) and hypovolemia with phenylephrine infusion (BW + PHE)

	**BL**	**BL + PHE**	**BW**	**BW + PHE**
SV (ml)	0.39 ± 0.04	0.27 ± 0.09	0.25 ± 0.08	0.19 ± 0.05^*^
AoF (mL ^**.**^ min^-1^)	71 ± 18	51 ± 15^*^	53 ± 13^*^	39 ± 10^*^
HR (bpm)	214 ± 26	198 ± 33	217 ± 33	201 ± 21
CVP (mm Hg)	3 ± 1	4 ± 1	3 ± 1	5 ± 1
MAP (mm Hg)	76 ± 16	77 ± 12	77 ± 16	81 ± 13
PAP (mm Hg)	38 ± 10	40 ± 15	32 ± 13	38 ± 13
LVEDP (mm Hg)	4.7 ± 5.7	5.7 ± 5.3	5.1 ± 4.7	4.2 ± 4.2
dP/dtmax (mm Hg ^**.**^ s^-1^)	1544 ± 456	1780 ± 946	1897 ± 1027	1838 ± 899
dP/dtmin (mm Hg ^**.**^ s^-1^)	-1802 ± 765	-1789 ± 631	-1763 ± 989	-1787 ± 1014

^*^p < 0.05 vs BL.

AoF: Aortic flow; CVP: Central venous pressure; dP/dtmax and dP/dtmin: Maximum and minimum first derivative of LVP, respectively; HR: Heart rate; LVEDP: Left ventricular end diastolic pressure; MAP and PAP: Mean and pulse aortic pressure, respectively; SV: Stroke volume.

**Figure 2:**
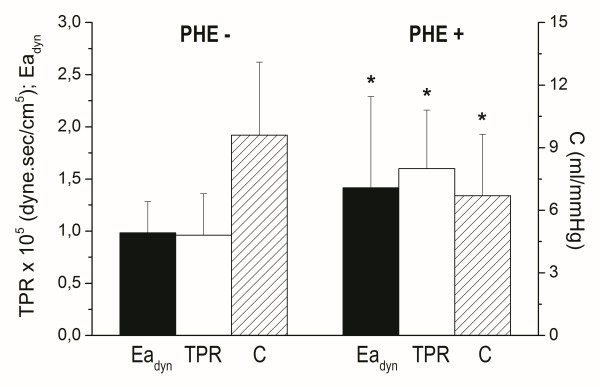
**Bar plot showing changes in arterial dynamic elastance (Ea**_**dyn**_**), total peripheral resistance (TPR), and systemic compliance (C) between baseline and hemorrhage (PHE-) and phenylephrine infusion (PHE+).** * *P* < 0.05.

Phenylephrine infusion induced significant increase in vasomotor tone (Figure 2) and it also induced a further decrease in aortic flow with a significant SV decrease (*P* < 0.05) (Table 1). Mean and pulse arterial pressure, and heart rate did not show significant changes. LV preload (estimated by LV end-diastolic pressure) and the maximum and minimum first derivative of LVP did not change during either hemorrhage and PHE infusion.

Baseline PPV, SPV, PPV_apnea_, and SVV increased significantly during hemorrhage (*P* < 0.05) (Table 2). However, after hemorrhage, PHE determined a decrease of all dynamic indices (*P* < 0.05), returning to baseline values (Table 2). There was a significant correlation between SVV and PPV, PPV_apnea_ and SPV during BL and BW (without PHE infusion) (r^2^ ≥ 0.5). However, there was no correlation between SVV and its pressure surrogates during PHE infusion.

**Table 2:** Dynamic indices data during normovolemia (BL), normovolemia with phenylephrine infusion (BL + PHE), hypovolemia (BW) and hypovolemia with phenylephrine infusion (BW + PHE)

	**BL**	**BL + PHE**	**BW**	**BW + PHE**
SVV (%)	12 ± 6	10 ± 4	32 ± 9^*§^	11 ± 5^‡^
PPV (%)	13 ± 6	12 ± 5	28 ± 10^*§^	15 ± 7^‡^
SPV (%)	10 ± 4	7 ± 3	19 ± 5^*§^	12 ± 5^‡^
PPV_apnea_ (%)	12 ± 6	11 ± 4	21 ± 7^*§^	14 ± 6^‡^

^*^p < 0.05 vs BL, ^§^p < 0.05 vs BL + PHE, ^‡^p < 0.05 vs BW.

PPV: Pulse pressure variation; PPV_apnea_: PPV using PP during apnea as denominator; SVV: Stroke volume variation; SPV: Systolic pressure variation.

**Figure 3:**
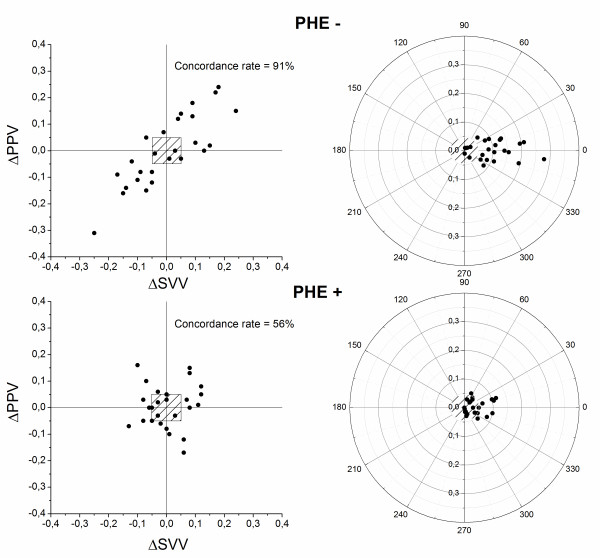
**The four-quadrant plots and polar plots to examine the trending abilities of PPV against SVV under normal vasomotor tone (PHE-) and under increase vasomotor tone (PHE+).** Half-circle polar plots are shown with data transformed to positive directional data only. We applied an exclusion zone when the percentage of change of data was below 15% (0.5). SVV: stroke volume variation, PPV: pulse pressure variation.

We used the four-quadrant plots and polar plots to examine the trending abilities of arterial pressure surrogate dynamic indices against SVV in normal and high vasomotor tone. In the four-quadrant plot analysis, we found that changes in PPV, PPV_apnea_ and SPV were 91%, 95% and 76% concordant with SVV in normal vasomotor tone, and significantly decreased by phenylephrine administration (56%, 53% and 43%, respectively) (Table 3 and Figure 3).

**Table 3:** **Polar analysis data between SVV and PPV, PPV**_**apnea**_**and SPV during experimental conditions without phenylephrine (PHE-; BL and BW) and with phenylephrine (PHE+; BL + PHE and BW + PHE)**

		**Concordance rate (%)**	**Mean angular bias (°)**	**Radial limits of agreement (°)**
	PPV	91	2.2	21
PHE-	PPV_apnea_	95	-3.2	21
	SPV	76	-4.1	26
	PPV	56	2.9	29
PHE+	PPV_apnea_	53	-5.1	19
	SPV	43	-14.1	26

PPV: Pulse pressure variation; PPV_apnea_: PPV using PP during apnea as denominator; SVV: Stroke volume variation; SPV: Systolic pressure variation. BL: Basal; BW: Blood withdrawal.

The polar plot analysis showed that mean angular bias was 2.2°, -3.2°, -4.1° (PHE-) and 2.9°, -5.1° and -14.1° (PHE+), and the radial limits of agreement were 21°, 21° and 26° (PHE-) and 29°, 19° and 26°, respectively (Table 3 and Figure 3).

## Discussion

The results from this rabbit hemorrhage model demonstrate that the infusion of phenylephrine (a pure α1-receptor agonist) blunts the dynamic preload indexes increase after bleeding. This effect is mainly due to an acute increase of vasomotor tone (decrease of arterial compliance and Ea_dyn_ and TPR increase) without any apparent change of the effective intravascular volume. This result may suggest the limitation of dynamic indexes in predicting fluid responsiveness in routine clinical practice during hypovolemia and vasopressor drug administration.

Several studies have shown that PPV, SPV and PPV_apnea_ are able to predict fluid responsiveness as surrogates of SVV [6,10,11]. Accordingly, in normal vasomotor tone (BL and BW) all dynamic arterial pressure indices changed in the same direction. Therefore, SVV correlated with its arterial pressure surrogates (Pearson's product moment r > 0.7) and showed a high concordance rate (quadrant plot analysis) and acceptable mean angular bias and radial limits of agreement (polar plot), except with SPV. However, the concordant rate in four-quadrant plot analysis was poor during PHE administration, and mean angular bias was higher than that in normal vasomotor tone. This means that the trending ability of the arterial pressure surrogates against SVV was not acceptable during hemorrhage under increased vasomotor tone.

In the present study, we used the gold standard reference method for CO measurement (ultrasonic flowprobe) that directly measures instantaneous aortic flow with a high degree of precision (± 1% to 2%). Hence, we could estimate SV and SVV with a high certainty, and allowed us to use SVV as a reference functional dynamic index [25]. Studies using technologies such as esophageal Doppler or pulse contour analysis (calibrated or uncalibrated) may be impacted by the device itself, decreasing the precision of the measurement of CO [26,27]. Also, the validity of algorithms using these devices to track actual SV in patients with altered arterial stiffness has shown a decrease of SVV for prediction of fluid responsiveness in patients [26,27]. Furthermore, we estimated the dynamic parameters manually and synchronized with the respiratory cycle which is highly accurate [5,6].

To date there are insufficient and contradictory data on the effect of vasoconstrictor therapy on dynamic preload indicators either in experimental and controlled condition or clinical setting [13-18]. Nouira et al. [13] have showed that norepinephrine (NE) induces a significant increase in CO and SV during severe hypovolemia with a concomitant decrease in PPV and SPV. They proposed that both effects were related to the shift of blood from unstressed to stressed blood volume. This is in accordance to Renner et al. [14] data on an animal hemorrhage model. However, on account of the global end-diastolic volume (PiCCO) remained unchanged, they proposed the effects of NE administration beyond shifting blood from unstressed to stressed blood volume. In an euvolemic model of ventilated pigs, Kubitz et al. [15] found that both SPV and PPV correlated with SVV (aortic Doppler flow probe). However, the correlations between SVV and SPV and PPV became weaker during the arterial pressure increase by PHE infusion. In the clinical setting, Sakka et al. [16] found that an increase in blood pressure reduces SVV significantly, without changing SPV in cardiac surgical patients. Wajima et al. [17] found that in patients scheduled to undergo elective surgery, induced hypertension (PHE) decrease SVV and induced hypotension (nitroglycerine) increased SVV. On the contrary, Hadian et al. [18] proposed that SVV and PPV may be used to guide fluid replacement algorithms in cardiac surgical patients receiving vasoactive drugs therapy. None of these studies analyzed in a separate manner the direct effects of a pure vasopressor versus the indirect effects of the change in arterial pressure on the arterial wall viscoelastic properties.

We demonstrate that the ability of the dynamic indexes to predict fluid responsiveness during hemorrhage under an isobaric increase of vasomotor tone (TPR and Ea_dyn_ increase and C decrease) induced by phenylephrine administration (a pure α1-agonist) would not be acceptable. The increase of SVV and its surrogates after bleeding was blunted under PHE infusion. The concomitant significant decrease of SV could also influence on this response. Arterial stiffness depends not only on intravascular pressure but also on viscoelastic properties of the vascular wall. The isobaric analysis (mean aortic pressures remained stable during BW and BW + PHE), avoids the indirect effect of the arterial pressure on the viscoelastic properties of the arterial wall [28]. Therefore, the significant TPR increase (steady component of afterload) and C decrease (pulsatile component of afterload) during BW + PHE are secondary to a direct effect of PHE on the viscoelastic properties of the arterial vascular wall. Accordingly, the PPV/SVV ratio, a functional approach to arterial tone assessment, showed a significant increase and could explain the absence of correlation and concordance rate between SVV and its surrogates during PHE infusion. The presence of both effects (direct and indirect) on arterial stiffness during vasoactive drugs therapy plus a central effect on the cardiac contractility by the use of α1 and β1 agonist (like NE), could explain some of the contradictory data referred previously.

α1-agonists have the ability to both increase (by reducing venous compliance, thus converting unstressed to stressed blood volume and increasing preload) and decrease (primarily through increases in venous resistance) CO [29]. The result is likely related to dose of the drug, the reserve in unstressed blood volume and the initial tone in the veins, all of which affect the recruitable blood volume, a critical hemodynamic reserve that cannot be measured by any monitoring device [30]. PHE creates countervailing effects increasing the stressed blood volume through the recruitment of unstressed volume in the reservoir compartment (which increase the venous return) and increasing the venous resistance through the mean radius decrease in the large veins (which decrease the venous return), concomitantly. Therefore, although stimulation of α1-agonist receptors decreases venous capacitance and may transiently increase venous return by recruitment of unstressed to stressed blood volume, this gain may be offset by the increase in venous resistance and afterload [29,31]. Although we cannot discard a reduction of the intrathoracic blood volume and/or the presence of right ventricular dysfunction during PHE infusion secondary to pulmonary hypertension, the non significant change of LV end-diastolic pressure would support a predominant increase of vasomotor tone.

Recently, Cannesson et al. demonstrated that the impact of PHE bolus on CO is related to the position of the heart on the Frank-Starling relationship and is dose dependent. They observed that when LV is preload independent, PHE induces a decrease in CO and viceversa, when it is preload dependent, PHE boluses induce an increase in CO [32]. On the contrary, our data showed that PHE infusion induced a further decrease in aortic flow with a significant SV decrease after blood withdrawal compared with normovolemia (BL + PHE), secondary to a significant increase of vasomotor tone and maintaining the mean arterial pressure. Probably, the higher dose and continuous infusion could explain the differences during hypovolemia. Regarding the pleth variability index (PVI), a non-invasive alternative to PPV and SVV, Monnet et al. reported that the prediction of fluid responsiveness by PVI is less reliable than invasive indexes in patients with acute circulatory failure receiving norepinephrine [33].

During BW, mean arterial pressure (CO × TPR) and pulse arterial pressure (SV/C) were maintained at the expense of a CO and SV decrease, secondary to a 45% increase of TPR and 14% decrease of C, probable due to the sympathetic modulation of the arterial tone. Accordingly, Monnet et al. comparing patients with acute circulatory failure who received a fluid challenge or in whom NE was introduced or increased, showed that changes in pulse arterial pressure could not be used for monitoring the effects of NE on CO [34]. Natalini et al. also showed that arterial pressure did not enable to predict the individual response to volume administration in a cohort of hypotensive patients receiving NE and dobutamine [35].

### Study limitations

Although, the arterial pressure and flow signal are acquired in the abdominal aorta, where the arterial wall stiffness and reflection wave are higher than proximal central aorta, we took the caution to obtain both measurements at the same site. It is widely accepted that peripheral pulse pressure depends mainly on SV and arterial stiffness (1/compliance). In this regard, the absence of pulse pressure changes during PHE with concomitant SV decrease is related to concomitant decrease of arterial compliance [36,37]. We only estimate the compliance of the abdominal aorta, not including ascending and descending aorta compliances. Nevertheless, because total compliance of a system is the sum of the individual compliances in series, the change of the abdominal aorta compliance could be representative of the compliance of the entire aorta [37].

Flow probe only measures descending aortic flow (about 70%) and excludes flow to the aortic arch vessels (30%). Although the assumption of a constant diversion of blood flow during PHE infusion may be violated, previous experimental animal data suggest that phenylephrine increases arteriolar resistance uniformly in the different vascular territories [29]. This fact plus the measure of blood pressure and flow at the same place could make the dynamic indexes estimated at abdominal aorta representative of the whole arterial circulation.

Although, the LV end-diastolic pressure maintenance could rule out a significant shift blood from unstressed to stressed blood volume, we cannot discard an increase of pulmonary arterial pressure secondary to PHE infusion, concomitantly [29].

## Conclusions

All dynamic preload indicators (SVV, SPV, PPV and PPV_apnea_) were significantly reduced by phenylephrine during hemorrhage, mainly by increasing the vasomotor tone. In this animal model of hemorrhage and increased vasomotor tone induced by phenylephrine the ability of dynamic indices to predict fluid responsiveness seems to be impaired, masking the true fluid loss. Moreover, the arterial pressure surrogates have not the reliable trending ability against SVV measured by ultrasound flowprobe. Consequently, in clinical routine practice, we should be aware that vasopressors can substantially reduce the ability of the dynamic indicators to predict fluid responsiveness, masking the effective intravascular volume deficit.

## Abbreviations

BL: Baseline condition; BW: Blood withdrawal; C: Arterial capacitance; CO: Cardiac output; dP/dtmax and dP/dtmin: Maximum and minimum first derivative of LV pressure; Eadyn: Dynamic elastance; LV: Left ventricle; NE: Norepinephrine; PHE: Phenylephrine; PPV: Pulse pressure variation; PPVapnea: PPV during apnea; SPV: Systolic pressure variation; SV: Stroke volume; SVV: SV variation; TPR: Total peripheral resistance.

## Competing interests

The authors declare that they have no competing interests.

## Authors’ contributions

JPB conceived of the study, participated in its design, conducted the study, analyze the data, and wrote the manuscript. JR participated in the design of the study and helped write the manuscript. JCG conceived of the study, participated in its design, conducted the study, analyze the data, and wrote the manuscript. All authors read and approved the final manuscript.

## Pre-publication history

The pre-publication history for this paper can be accessed here:

http://www.biomedcentral.com/1471-2253/13/41/prepub
